# *Trans*-nuclei CRISPR/Cas9: safe approach for genome editing in the edible mushroom excluding foreign DNA sequences

**DOI:** 10.1007/s00253-024-13367-0

**Published:** 2024-12-30

**Authors:** Daishiro Koshi, Junko Sugano, Fuga Yamasaki, Moriyuki Kawauchi, Takehito Nakazawa, Minji Oh, Yoichi Honda

**Affiliations:** 1 Graduate School of Agriculture, Kyoto University, Sakyo-Ku, Kitashirakawaoiwakecho, Kyoto 606-8502 Japan; 2 Mushroom Research Division, Rural Development Administration, National Institute of Horticultural and Herbal Science, Bisanro 92, Eumseong, Chungbuk 27709 Republic of Korea

**Keywords:** Agaricomycete, Mushroom, CRISPR/Cas9, Non-GMO, *Pleurotus ostreatus*

## Abstract

**Abstract:**

Clustered regularly interspaced short palindromic repeat (CRISPR)/CRISPR-associated protein 9 (Cas9)-assisted genome editing has been applied to several major edible agaricomycetes, enabling efficient gene targeting. This method is promising for rapid and efficient breeding to isolate high-value cultivars and overcome cultivation challenges. However, the integration of foreign DNA fragments during this process raises concerns regarding genetically modified organisms (GMOs) and their regulatory restrictions. In this study, we developed a foreign-DNA-free genome editing method in *Pleurotus ostreatus* by transferring the Cas9/guide RNA (gRNA) complex between nuclei in the dikaryotic state. We isolated a donor monokaryotic *P. ostreatus* strain expressing Cas9 and gRNA targeting *pyrG* by introducing a recombinant plasmid, which exhibited uracil auxotrophy and 5-fluoroorotic acid (5-FOA) resistance. This strain was then crossed with a *pyrG*^+^ recipient monokaryon, resulting in dikaryotic strains exhibiting 5-FOA resistance after mycelial growth. When these strains were de-dikaryonized into monokaryons through protoplasting, we obtained monokaryotic isolates harboring the recipient nucleus with small indels at the *pyrG* target site. Importantly, these isolates were confirmed to be free of foreign DNA through genomic PCR, Southern blotting, and whole-genome resequencing analyses. This is the first report of an efficient genome editing protocol in agaricomycetes that ensures no integration of exogenous DNA. This approach is expected to be applicable to other fungi with a dikaryotic life cycle, opening new possibilities for molecular breeding without the concerns associated with GMOs.

**Key points:**

• *Successful genome editing via CRISPR/Cas9 trans-nuclei manner in P. ostreatus*.

• *Recipient monokaryons from gene-edited dikaryons showed no exogenous DNA sequences*.

• *Efficient genome editing protocol for safer molecular breeding in mushroom fungus*.

**Graphical Abstract:**

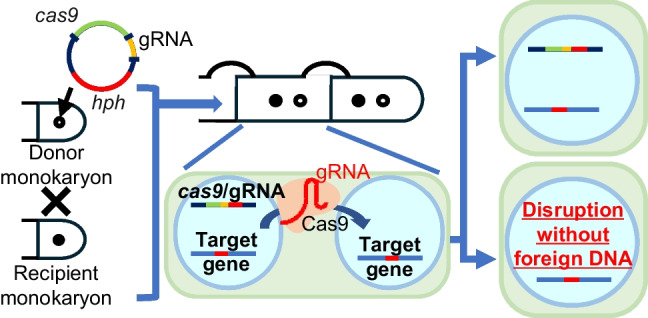

**Supplementary information:**

The online version contains supplementary material available at 10.1007/s00253-024-13367-0.

## Introduction

Numerous edible mushroom species, such as *Agaricus bisporus* (button mushroom), *Pleurotus* spp. (oyster mushrooms), *Lentinus edodes* (shiitake mushroom), *Auricula auricula* (wood ear mushroom), *Flammulina velutipes* (winter mushroom), and *Volvariella volvacea* (straw mushroom), are cultivated on an industrial scale (Chang 1999; Sánchez 2004). Mushroom production has grown substantially since 2000, with continued market expansion expected (El Sheikha and Hu 2018). Consequently, efficient breeding techniques are essential for developing high-value strains and overcoming cultivation challenges (Sonnenberg et al. 2017; Barh et al. 2019). For instance, spore dispersal in oyster mushrooms can cause allergies in cultivators and equipment damage (Baars et al. 2000), prompting research into sporeless strains (Eger et al. 1976; Obatake et al. 2003; Joh et al. 2004; Lavrijssen et al. 2020; Yamasaki et al. 2022).

Modern mushroom breeding increasingly emphasizes molecular techniques, modifying genes identified through whole-genome sequencing and bioinformatics (Dong et al. 2022; Nakazawa et al. 2024). For instance, creating a non-homologous end joining (NHEJ)-deficient *P. ostreatus* strain through disrupting the *ku80* gene has enabled efficient gene targeting, leading to sporeless strains (Salame et al. 2012; Yamasaki et al. 2021). However, these molecular breeding techniques, relying on foreign DNA integration for gene modification, as well as for transformant screening, result in genetically modified organisms (GMOs). GMOs face international regulations due to potential biodiversity threats (Gupta and Falkner 2006), limiting molecular breeding applications.

A potential solution is genome editing using clustered regularly interspaced short palindromic repeats (CRISPR)/CRISPR-associated protein 9 (Cas9), an adaptive immune system found in archaea and bacteria (Ishino et al. 1987; Jinek et al. 2012). This versatile technique, compared to that of conventional gene targeting methods, allows for its application across a wide range of species (Song et al. 2019). The method guides the Cas9 endonuclease to a target site using a 20-nt guide RNA (gRNA), leading to double-stranded DNA cleavage activating potential mutations through error-prone NHEJ repair. Theoretically, this mutation process requires only Cas9 and gRNA, without foreign DNA insertion. While the GMO status of such edits is debated, many countries have opted not to regulate them as GMOs (Buchholzer and Frommer 2023). Some genome-edited crops such as canola and soybean in the USA, and tomato and multiple species of fish in Japan, have already been approved as non-GMOs and are commercially farmed for human consumption (Menz et al. 2020; MAFF 2024; USDA 2024).

In recent years, CRISPR/Cas9 has been applied to edible mushrooms, including *A. bisporus*, *L. edodes*, *Flammulina filiformis*, and *P. ostreatus* (Moon et al. 2021; Boontawon et al. 2021a; Liu et al. 2022; Choi et al. 2023).

There are two broad approaches for CRISPR/Cas9-based genome editing: DNA-based and RNP (ribonucleoprotein)-based methods. In the DNA-based approach, plasmids containing Cas9 and gRNA expression sequences are introduced into host cells to achieve genome editing. However, this method often results in the integration of foreign plasmid DNA into the host chromosomes, hindering the development of non-GMO genome editing (Nakazawa et al. 2024). Our previous research achieved marker-free genome editing using transient transformation for CRISPR/Cas9 expression in *P. ostreatus* and *Gelatoporia subvermispora*; although, frequent insertions of partial foreign DNA fragments derived from the introduced plasmid were observed (Nakazawa et al. 2022; Koshi et al. 2022).

In contrast, the RNP-based approach involves the direct introduction of pre-assembled Cas9/gRNA complexes into cells, eliminating the need for encoding DNA fragments. However, this method currently lacks effective screening systems and may be less efficient than the DNA-based approach (Boontawon et al. 2021b). To address this issue, an RNP-dependent CRISPR/Cas9 system targeting double genes (one for screening and the other as the target gene) was developed in *P. ostreatus* (Boontawon et al. 2023). In *Ganoderma lucidum*, however, the integration of foreign DNA sequences derived from *Escherichia coli* and the Cas9 expression vector is observed in RNP-driven genome-edited strains. This contaminant DNA is likely due to residual DNA not being properly removed during Cas9 protein purification processes (Eom et al. 2023). Given these challenges, it is essential to develop an efficient and convenient non-GM genome editing method for mushrooms.

Many cultivated mushrooms are agaricomycetes with dikaryotic life cycle stages. In this stage, dikaryotic hyphae result from the mating of two haploid monokaryons with different mating types, where the two nuclei from the mating partners coexist in each cell without fusing and continue to grow vegetatively (Kamada 2002). When subjected to protoplasting, some of the dikaryotic hyphae can be re-separated into monokaryons (Larraya et al. 1999; Irie et al. 2001). Utilizing this feature by crossing a target monokaryon with another monokaryon harboring a Cas9/gRNA expression fragment, followed by re-separation, might allow genome editing without the insertion of foreign DNA sequences in the target monokaryon.

In this study, we developed a method for genome editing without foreign DNA integration in *P. ostreatus* by utilizing the migration of Cas9 and gRNA between nuclei in the dikaryotic state (i.e., *trans*-nuclei CRISPR/Cas9, see Graphical abstract). To our knowledge, this study represents the first report of an efficient CRISPR/Cas9 system in agaricomycetes that ensures the absence of foreign DNA in genome-edited strains.

## Materials and methods

### Strains, media, culture conditions, and genetic manipulation

The monokaryotic strain PC9 of *P. ostreatus* (Larraya et al. 1999; Spanish Type Culture Collection accession number CECT20311) and a wild-type monokaryotic strain #61 (this study; NITE Biological Resource Center accession number NBRC116971) were used for transformation. Details of the strains used in this study are listed in Supplemental Table S1. Strains were cultured on yeast extract, malt extract, and glucose (YMG) medium (Rao and Niederpruem 1969) solidified with 1.5% (w/v) agar and incubated at 28°C in continuous darkness. For the cultivation of *pyrG* mutants, YMG agar plates were supplemented with 20 mM uridine and 0.18 mM uracil (YMGUU). YMGUU plates containing 0.1% (w/v) 5-fluoroorotic acid (5-FOA) or 100 µg/ml hygromycin B were used to test for resistance and sensitivity to these antibiotics (Salame et al. 2012; Nakazawa et al. 2016).

PEG/CaCl_2_ transformation was performed using protoplasts prepared from mycelial cells, following the protocol by Salame et al. (2012). For selecting transformants with hygromycin B resistance, YMGUU plates supplemented with 100 µg/ml hygromycin B (YMGUU-Hyg) were used. Isolation of monokaryotic clones from the genome-edited dikaryon was done as described by Larraya et al. (1999) and Irie et al. (2001). Protoplasts diluted with 0.5 M sucrose were plated on YMGUU agar medium supported osmotically by 0.5 M sucrose.

### Design of pyrG-targeting sequences in gRNA

**Fig. 1:**
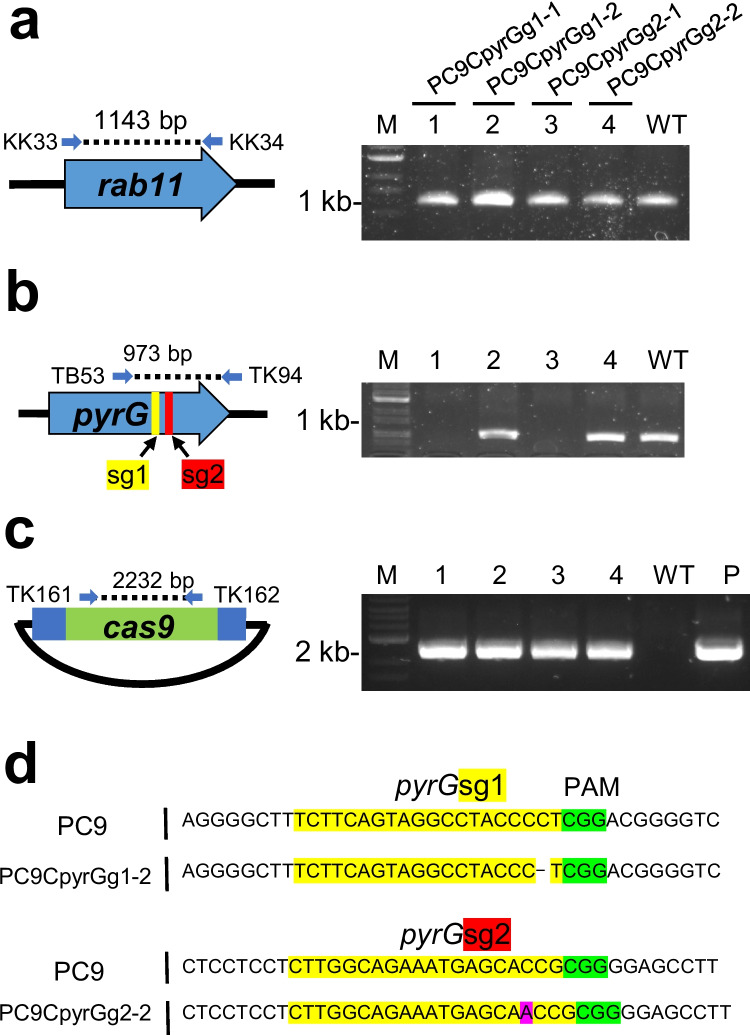
Acquisition of donor monokaryon strains using the PC9 strain. Genomic PCR amplification of **a** *rab11*, **b** *pyrG*, and **c** *cas9* in the transformants. Dashed lines in the schematic diagram indicate the regions amplified by genomic PCR. Blue arrows indicate the primer pairs used for the PCR experiment. Lane M: 1-kb ladder marker; lanes 1–4: the donor strains used in this study; lane WT (wild-type): wild-type PC9 strain; lane P: plasmid control. **d** Sequence analysis results of the *pyrG* fragment from (**b**)

Guide RNAs (gRNAs) targeting *pyrG* at nucleotide positions 91–111 and 521–540 from the putative start codon were designed by Boontawon et al. (2021a) (*pyrG*sg1 and *pyrG*sg2). The schematic of the *pyrG* gene and target site is shown in Fig. 1b, and the primers used for PCR are listed in Supplemental Table S2.

### Plasmid construction

Each gRNA was prepared by annealing two synthesized DNA oligos, TB41/TB42 and TB43/TB44, containing the *pyrG*-targeting sequences in *pyrG*sg1 and *pyrG*sg2, respectively (Supplemental Table S2). These oligos were then separately inserted into the *Bsa*I site of the linearized *cas9*-containing pCcPef3-126 plasmid (Supplemental Figure S1) (Sugano et al. 2017) using Golden Gate cloning (Engler et al. 2008). The resulting plasmids were designated pCcPef3-pyrGsg1 and pCcPef3-pyrGsg2. The primers used for plasmid construction are listed in Supplemental Table S2.

### Rapid genomic polymerase chain reactions

Genomic DNA extraction was performed as described by Izumitsu et al. (2012), followed by genomic PCR using KOD FX Neo (TOYOBO, Tokyo, Japan). To confirm genome extraction, an endoplasmic reticulum gene, *rab11*, which is unrelated to the target gene, was also amplified using primers KK33/KK34 as shown in Supplemental Table S2.

### Mating of donor and recipient strains, and selection

Mating of each donor strain (PC9CpyrGg1 and PC9CpyrGg2) with the recipient strain (#61) was conducted as described by Makino and Kamada (2004) on YMGUU agar plates (*ø* 9 cm). A small agar block (2 × 2 mm) with donor mycelium was inoculated at the center of a plate and incubated for 2–3 days until the mycelium diameter reached 1 cm. The donor mycelium in the centre of the colony was then replaced with a 2 × 2 mm inoculum from the recipient strain. After 3 or 6 days of incubation, the presence or absence of dikaryon formation was assessed by observing characteristic clamp cell formation at the edge of the mycelia under a light microscope.

**Fig. 2:**
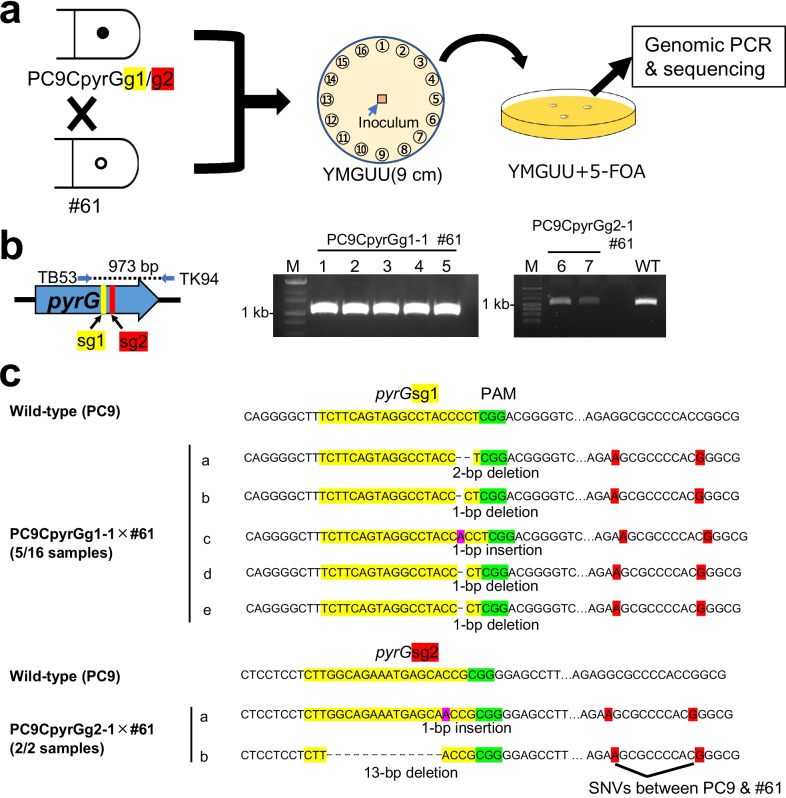
Confirmation of *trans*-nuclei genome editing. **a** Schematic representation of the experiment examining 5-FOA resistance and target site mutations in dikaryotic strains obtained by mating donor and recipient strains and collecting 16 mycelial samples along the edge of the plate. **b** Gel electrophoresis of genomic PCR amplifying the *pyrG* fragment in the 5-FOA resistant dikaryotic strains. The dashed line in the schematic diagram indicates the region amplified by genomic PCR. Blue arrows indicate the primer pairs used for the PCR experiment. Lane M: 1-kb ladder marker; lanes 1–5: five out of 16 strains derived from PC9CpyrGg1-1 × #61; lanes 6 and 7: two strains derived from PC9CpyrGg2-1 × #61; lane WT: wild-type dikaryotic strain from the PC9 × #61 cross. **c** Sequence analysis results of 5-FOA resistant dikaryotic strains obtained in Table 1. The shaded regions in the nucleotide sequence indicate yellow for the target sites of gRNA, green for protospacer adjacent motif (PAM) sequences, and red for single-nucleotide variants (SNVs) between PC9 and #61 strains. The results of genomic PCR for *pyrG* in 5-FOA resistant strains are shown in Supplemental Figure S1

The dikaryotic mycelium was sampled at 16 evenly distributed points from the plate’s edge and inoculated onto YMGUU agar plates containing 0.1% w/v 5-FOA (Fig. 2a).

### Southern blotting

Genomic DNA was extracted from mycelia of each strain cultivated on YMGUU liquid medium using the cetyl trimethyl ammonium bromide (CTAB) method, as described by Zolan and Pukkila (1986) and Muraguchi et al. (2003). The extracted DNA (approximately 3 µg) was then used for Southern blotting as described by Nakazawa et al. (2022). Each probe for *hph* and *cas9* was prepared by PCR amplification from pCcPef3-126 using primer sets TK203/TK204 and TK161/TK162, respectively. The primers used are listed in Supplemental Table S2.

### Whole-genome resequencing and bioinformatics

Genomic DNA was extracted from mycelia of the isolated monokaryon strains (RMg1b-1/2/3, RMg2a-1/2/3) and the #61 wild-type strain cultivated on YMGUU liquid medium using the CTAB method (Zolan and Pukkila 1986; Muraguchi et al. 2003). The resulting genomic DNA was subjected to whole-genome resequencing using NovaSeq6000 (Illumina, San Diego, USA; 150-bp paired-end sequencing). Sequence data are available in the DDBJ databases under the DRA accession number listed in Supplemental Table S3. Mapping of the obtained sequence reads to the reference sequence (PC9: http://genome.jgi.doe.gov/PleosPC9_1/PleosPC9_1.home.htm) was performed by QIAGEN CLC Genomics Workbench 23.0.5. The *k*-mers (*k* = 20) on both strands for all the extracted reads were investigated in the pCcPef3-pyrGsg1/sg2 sequences as described by Itoh et al. (2020) (https://github.com/taitoh1970/kmer). The statistical differences at the 1% level (*G* = 6.634) in read counts between the isolates and the wild-type strain were examined using a *G*-test for independence assuming model II (Sokal and Rohlf. 1970; Itoh et al. 2020).

## Results

### Isolation of donor monokaryon expressing Cas9 and gRNA

The *pyrG* gene, whose mutation confers resistance to 5-FOA and pyrimidine auxotrophy (Boontawon et al. 2021a), was selected as the CRISPR/Cas9 target site. Resistance to 5-FOA and pyrimidine auxotrophy is expected when the *pyrG* gene(s) is non-functional in the host strain. In the dikaryotic state, two nuclei coexist in a single cell, and disruption of *pyrG* in both nuclei is required to confer resistance to 5-FOA.

To isolate a monokaryotic strain expressing Cas9 and gRNA, the recombinant plasmid pCcPef3-pyrGsg1 or pCcPef3-pyrGsg2 was introduced into the *P. ostreatus* PC9 strain via protoplast-mediated transformation. These pCcPef3-126-based plasmids express *pyrG-*targeting gRNAs (*pyrG*sg1 and *pyrG*sg2) along with Cas9 and hygromycin phosphotransferase (Hph), conferring resistance to hygromycin B (Hyg) (Boontawon et al. 2021a). Totally 23 transformants grown on YMGUU-Hyg regeneration medium containing 0.5 M sucrose were transferred on fresh YMGUU plate containing 100 µg/ml of Hyg. Among them, 22 strains showed Hyg resistance and were further transferred on YMGUU-5-FOA medium, resulting in 15 5-FOA-resistant strains. From these, four strains harboring the *cas9* gene and a mutation at the *pyrG* target site were selected through genomic PCR and sequencing (Fig. 1). As a control, an unrelated gene, *rab11*, was amplified to confirm successful genome extraction from all samples (Fig. 1a). These strains were designated as donor strains PC9CpyrGg1-1, -2 and PC9CpyrGg2-1, -2, obtained by introducing pCcPef3-pyrGsg1 and pCcPef3-pyrGsg2, respectively. Genomic PCR of the *pyrG* sequence showed no fragment amplification in PC9CpyrGg1-1 and PC9CpyrGg2-1, suggesting large-scale mutations near the target site (Fig. 1b). Sequence analysis of the amplified fragments from PC9CpyrGg1-2 and PC9CpyrGg2-2 indicated small indels within the target site (Fig. 1d). These four strains were used as donor monokaryons for subsequent experiments.

### Genome editing across two nuclei in the crossed dikaryon

To confirm genome editing via Cas9/gRNA transfer between two nuclei in the dikaryotic state, each donor strain was crossed with the recipient wild-type compatible monokaryotic strain #61, as described in the “Materials and methods”. Crossed dikaryons were inoculated at the center of YMGUU plates, and after growth to the edge, 16 specimens were isolated from the plate edge. The presence of 5-FOA resistance was tested on YMGUU plates containing 0.1% w/v 5-FOA (Fig. 2a). Multiple 5-FOA resistant dikaryotic strains were obtained in all combinations of parental monokaryons (Table 1). When PC9CpyrGg1-1 or -2 was used as a donor, 5-FOA resistance was observed in 37.5% to 100% of the 16 samples. In contrast, when PC9CpyrGg2-1 or -2 was used, efficiencies ranged from 12.5 to 25.5%, except for the experimental replicate 2 (Rep 2) of PC9CpyrGg2-1x#61 where no resistant strains were observed (Table 1). In previous studies, *pyrG*sg1 was more efficient than *pyrG*sg2 in isolating 5-FOA resistant strains using CRISPR/Cas9 in the monokaryotic host strain PC9 in both plasmid-based and RNP-based methods (Boontawon et al. 2021a, b).

**Table 1:** Number of 5-FOA resistant strains observed in dikaryotic strains obtained by cross between donor and recipient strains

Dikaryon	Number of 5-FOA-resistant dikaryons		
Donor × recipient	Rep 1^a^	Rep 2	Rep 3
PC9CpyrGg1-1 × #61	16 (100%)	16 (100%)	15 (93.8%)
PC9CpyrGg1-2 × #61	6 (37.5%)	10 (62.5%)	12 (75.0%)
PC9CpyrGg2-1 × #61	2 (12.5%)	0 (0%)	2 (12.5%)
PC9CpyrGg2-2 × #61	2 (12.5%)	3 (18.8%)	4 (25.0%)

^a^Rep1-3 represents three times independent experiments

To investigate whether the *pyrG* gene in the recipient nucleus was edited in these dikaryotic strains, five strains from PC9CpyrGg1-1 × #61 and two strains from PC9CpyrGg2-1 × #61 were randomly selected from Rep 1. The *pyrG* region containing the gRNA target site was amplified by genomic PCR (Fig. 2b), and the fragments were sequenced. Since PC9CpyrGg1-1 and PC9CpyrGg2-1 have large-scale mutations in *pyrG* with no target site amplification in genomic PCR (Fig. 1b), only the recipient #61-derived *pyrG* was expected to be amplified by performing genomic PCR on the dikaryotic 5-FOA resistant strains. Mutations at the target site were confirmed in all sequenced strains (Fig. 2c). All mutations consisted of small indels, typically a few base pairs, except for PC9CpyrGg1-2 × #61 isolate b which exhibited a 13-bp deletion. The sequences were confirmed to be derived from the recipient #61 genome by comparing single-nucleotide variants (SNVs) with the PC9 genome (Fig. 2c, red highlights). Furthermore, these mutations are located at the target site of each gRNA sequence used in the experiment. These gRNA-dependent small indel mutations indicate that genome editing mediated by Cas9/gRNA transfer between the two nuclei occurs.

### Isolation of the recipient monokaryon from the genome-edited dikaryotic strains

**Fig. 3:**
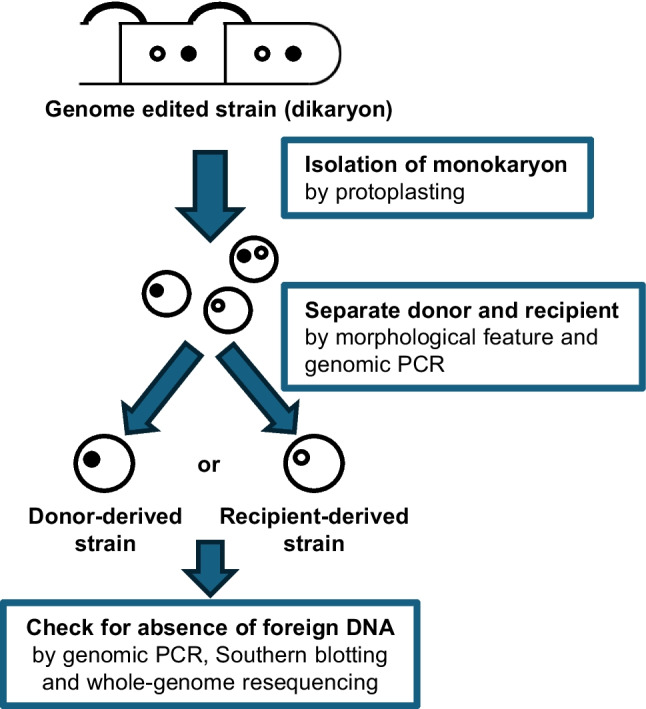
Schematic representation of the experiment isolating monokaryotic strains without foreign DNA by protoplasting

**Fig. 4:**
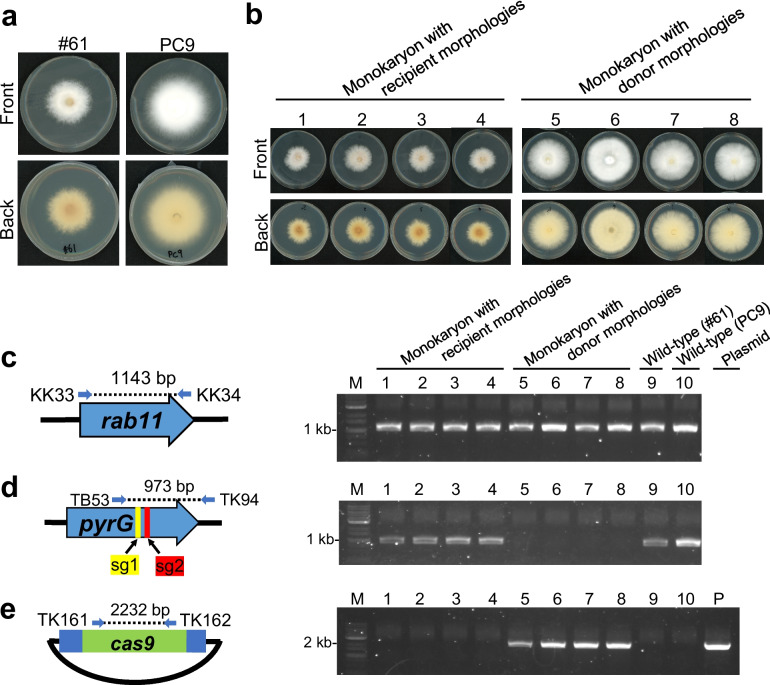
Isolation of monokaryotic strains without foreign DNA. **a** Growth photographs of #61 (left) and PC9 (right) strains after 7 days of culture. **b** Growth photographs of the isolated monokaryons after 7 days of culture. Genomic PCR amplification of **c** *rab11*, **d** *pyrG*, and **e** *cas9* in the isolates. Dashed lines in the schematic diagram indicate the regions amplified by genomic PCR. Blue arrows indicate the primer pairs used for the PCR experiment. Lane M: 1-kb ladder marker; lanes 1–4: monokaryons with #61 (recipient) morphologies; lanes 5–8: monokaryons with PC9 (donor PC9CpyrGg1/2–1) morphologies; lane 9: wild-type #61 strain; lane 10: PC9 strain; lane P: plasmid control

To obtain foreign DNA-free genome-edited monokaryons, monokaryons with recipient-derived nuclei were isolated from protoplasts of PC9CpyrGg1-1 × #61 isolates b and c, and PC9CpyrGg2-1 × #61 isolates a and b, followed by regeneration (Fig. 2c, Fig. 3). The PC9 and #61 strains can be distinguished by their morphology and growth rate: PC9 grows faster with more aerial hyphae, while #61 shows a darker colour on the reverse side (Fig. 4a). From each dikaryotic strains, four monokaryotic colonies with donor-derived morphologies and four with recipient-derived morphologies were successfully isolated (Fig. 4b). Each monokaryotic strain was checked for the presence of a donor or recipient nucleus through genomic PCR amplification of the *pyrG* fragment (Fig. 4d, Supplemental Figure S2). As a control, the unrelated *rab11* gene was amplified to confirm successful genome extraction from all samples (Fig. 4c, Supplemental Figure S2). In donor morphology strains, no amplification of *pyrG* was observed due to the large-scale mutation as in the original donor strains (Figs. 1b and 4d). In recipient morphology strains, the *pyrG* fragment was successfully amplified, and sequence analysis confirmed that all isolates preserved the mutations at the target site (Supplemental Figure S3). Genomic PCR targeting the *cas9* DNA sequence revealed no band in recipient-derived monokaryons, whereas an amplification band was observed in donor-derived monokaryons (Fig. 4e, Supplemental Figure S2). These results suggest that the isolates with recipient-derived nuclei did not contain the *cas9* expressing plasmid fragment.

### No plasmid-derived sequence was detected in the recipient-derived monokaryons

To confirm the absence of foreign DNA, six recipient-derived monokaryons (RM) from PC9CpyrGg1-1 × #61 isolate b (designated as RMg1b-1, −2, −3) and PC9CpyrGg2-1 × #61 isolate a (designated as RMg2a-1, −2, −3) were analysed by Southern blotting with probes for a part of *hph* and *cas9*. Specific DNA fragments for both *hph* and *cas9* were detected in the original donor strains PC9CpyrGg1-1 and PC9CpyrGg2-1, but not in the six genome-edited monokaryons (Supplemental Figure S4).

For further confirmation of the presence or absence of foreign DNA, whole-genome resequencing was performed with these six isolates and the obtained read data confirmed sufficient coverage (Supplemental Table S3). For the detection of plasmid-derived sequences from these read data, *k*-mer analysis was adapted as it has been reported to be an effective method for detecting foreign DNA in several major genome-edited crops (Itoh et al. 2020; Yasumoto and Muranaka 2023). In general, an insertion of extremely short DNA fragments cannot be distinguished from endogenous regions and can be excluded from risk assessments for non-GMO (Itoh et al. 2020). In contrast, DNA fragments longer than 20 bp are reported to be unique and distinguishable (Lusser et al. 2011). Furthermore, *k*-mer analysis has been reported to detect foreign DNA inserts of 20 bp or longer with a very low false positive rate when the sequencing depth is sufficiently high (above × 30) in multiple plant species (Itoh et al. 2020). Therefore, *k*-mer analysis with *k* = 20 was performed, counting the number of reads that matched every 20 bp of the vector sequence.

**Fig. 5:**
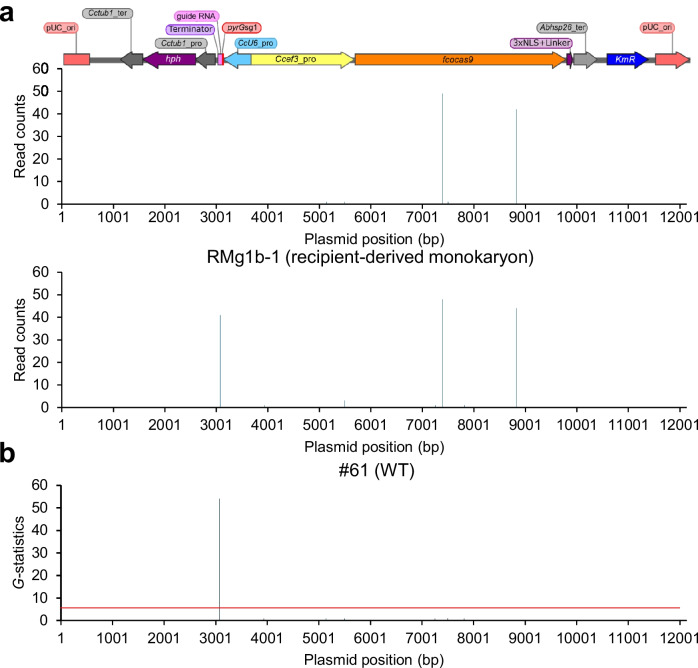
Detection of identical 20-mers between the genome of the isolated monokaryon with nuclei derived from #61 and vector sequences. The *y*-axis show **a** the read counts and **b** the *G*-statistic of a 20-mer at each nucleotide position. The *x*-axis indicates the nucleotide positions on a vector sequence. The red horizontal line corresponds to the 1% significance level (*G*-values > 6.634). pUC_ori, origin for *Escherichia coli*; *kmR*, kanamycin resistance gene; *Abhsp26*_ter, terminator from *hsp26* gene in *Agaricus bisporus*; NLS, nuclear localization signal; *fcocas9*, fungal and plant codon optimized Cas9 (Sugano et al. 2017); *Ccef3*_pro, promoter from *ef3* gene in *Coprinopsis cinerea*; *CcU6*_pro, *U6* promoter from *C. cinerea*; *Cctub1*_pro or ter, promoter or terminator from *tub1* gene in *C. cinerea*; *hph*, hygromycin resistance gene

Two major peaks in the isolates and three in the wild-type #61 strain were detected in the read count data (Fig. 5a, Supplemental Figure S5). The two peaks in the isolates matched those in the wild-type strain, indicating they were not plasmid-derived sequences. A *G*-test for independence identified one significant peak in the wild-type strain, with no other significant peaks detected (Fig. 5b, Supplemental Figure S6). This peak corresponds to the 20 bp gRNA target sequence in the wild-type *pyrG* genome, and its absence explains the mutations at the target site in the isolates. These results demonstrate that the genome-edited monokaryons via *trans*-nuclei CRISPR/Cas9 do not contain foreign plasmid DNA.

## Discussion

Recently, efficient plasmid-based CRISPR/Cas9 systems have been reported in several agaricomycetes, including *Coprinopsis cinerea* (Sugano et al. 2017), *G. lucidum* (Qin et al. 2017), *L. edodes* (Moon et al. 2021), *G. subvermispora* (Nakazawa et al. 2022), and *P. ostreatus* (Boontawon et al. 2021a). However, these systems often rely on plasmid integration for CRISPR/Cas9 expression, raising concerns about increased off-target effects and unforeseen adverse impacts due to constitutive expression. Previous studies from our group have explored transient transformation-based CRISPR/Cas9 in *G. subvermispora* and *P. ostreatus*, but partial integration of plasmid fragments was observed in genome-edited isolates (Nakazawa et al. 2022; Koshi et al. 2022). In this study, we present a novel approach, *trans*-nuclei CRISPR/Cas9, which successfully achieves genome editing without foreign DNA insertion. This method offers a plasmid-free alternative for genome editing in agaricomycetes and has potential for application in many other fungi with a dikaryotic life cycle.

Our study provides the first demonstration of genome editing in basidiomycetes through the transfer of Cas9 and gRNA between nuclei within dikaryotic cells. These findings also offer direct evidence that exogenous recombinant proteins with nuclear localization signals can move between nuclei and function in both, even when expressed from a single nucleus. Extensive research suggests that the transcriptional regulator HD1 may cooperate with HD2 from different nuclei to activate the *A* mating type pathway in dikaryotic cells (Raudaskoski and Kothe 2010; Kües 2015). These observations raise intriguing questions about the nature of interactions between the two nuclei in dikaryotic cells, including the movement of proteins and small RNAs.

During the growth of dikaryotic isolates composed of donor and recipient nuclei, *trans*-nuclei CRISPR/Cas9 may occur occasionally. Once a mutation is generated, no further attack by Cas9/gRNA may occur in the nucleus due to a mismatch between the mutated target site and gRNA. The mutations found in the edited strains varied (Fig. 2c) even though they were isolated from the same plate (Fig. 2a), suggesting that *trans*-nuclei genome editing happened independently during the growth of a colony. It is plausible that within a dikaryotic colony, there are various recipient-derived nuclei, including wild-type and ones containing a mutation at the target site. This kind of heterozygosity was resolved in the monokaryon isolating process through protoplasting and regeneration, which may serve as a purification process for each edited nucleus to isolate a genetically pure strain. Although the recipient nucleus can be picked up through di-mon mating using a suitable compatible monokaryon, the protoplast regeneration protocol used in the present work may be better for isolating a genetically pure edited strain, as strains obtained through di-mon mating contain both the recipient nucleus with the target gene disrupted and another nucleus without disruption, making them potentially less ideal for molecular breeding.

Investigation of gRNA efficiency in *trans*-nuclei CRISPR/Cas9 disruption revealed that *pyrG*sg1 (37.5–100%) outperformed *pyrG*sg2 (0–25%) in generating 5-FOA-resistant strains (Table 1). This aligns with previous findings by Boontawon et al. (2021a) using the PEG/CaCl_2_ method, where efficiencies ranged from 47.1 to 94.7% for *pyrG*sg1 and 28.6% for *pyrG*sg2 with pCcPef3-126-pyrGsg1/sg2. These results suggest that the efficiency of *trans*-nuclei genome editing, like plasmid-mediated editing in monokaryons, is highly dependent on the gRNA used. Notably, using donor strain PC9CpyrGg1-1 yielded near 100% efficiency in all three attempts, while PC9CpyrGg1-2 resulted in efficiencies of 37.5% to 75%. This variation may be due to differences in integration sites and copy numbers of introduced plasmids among donor strains, resulting in varying expression levels of Cas9/gRNA. For highly effective *trans*-nuclei CRISPR/Cas9, pre-selecting of high-efficiency donor strains could be crucial. In this study, genome-edited strains were obtained by *trans*-nuclei CRISPR/Cas9 targeting the marker gene *pyrG*, which allows for selection using 5-FOA. Such selection is not possible for most other endogenous genes as a target of genome editing. In this case, highly efficient *trans*-nuclei CRISPR/Cas9 would be required for successful isolation of edited strains. Future research should investigate how this method can be adapted for general gene editing.

This study further succeeded in isolating plasmid-free genome-edited monokaryons from the strains that underwent *trans*-nuclei genome editing. For genome-edited crops, there is an increasing emphasis on investigating the absence of foreign DNA in the genome to reduce risks to human health (Buchholzer and Frommer 2023). Several groups support the notion that a comprehensive investigation involving multiple methods, including whole-genome sequencing (WGS) is effective (Itoh et al. 2020; Yasumoto and Muranaka 2023). Using genomic PCR, Southern blotting, and WGS, we rigorously demonstrated that these strains are free of foreign DNA. Detection of foreign DNA from WGS data was performed using* k*-mer analysis, which does not rely on reference sequences. This method has been demonstrated as an effective detection method in major crops like rice and potatoes (Itoh et al. 2020; Yasumoto and Muranaka 2023). To the best of our knowledge, this is the first time such a thorough detection test for foreign DNA has been achieved in genome-edited agaricomycetes. Although not performed in this study, it is expected that assembling a reference genome for the wild-type strain #61 and aligning it with sample reads would similarly allow for comprehensive detection of foreign DNA, while also facilitating further investigation into the effects of off-target activity.

In conclusion, we demonstrated a genome editing method in *P. ostreatus* utilizing the *trans*-nuclei delivery of Cas9/gRNA in the dikaryotic state. This study is the first to achieve genome editing in filamentous fungi using plasmid-based CRISPR/Cas9 without the insertion of foreign DNA fragments. This method offers promising potential for safe and efficient genome editing-based breeding in filamentous fungi with a dikaryotic state.

## Supplementary information

Below is the link to the electronic supplementary material.

Supplementary file1 (PDF 1082 KB)

## Data Availability

Whole-genome resequencing data for the genome of the isolated monokaryon strains (RMg1b-1/2/3, RMg2a-1/2/3) were submitted to the DDBJ Sequence Read Archive (DRA) and available in the database at the DNA Data Bank of Japan (DDBJ).
