# Supplementary material for: Trans-nuclei CRISPR/Cas9: safe approach for genome editing in the edible mushroom excluding foreign DNA sequences
Source: Appl Microbiol Biotechnol. 2024 Dec 30;108:548. doi: 10.1007/s00253-024-13367-0 (PMC11685257; doi:10.1007/s00253-024-13367-0)
Supplement: Supplementary file 1 [file 253_2024_13367_MOESM1_ESM.pdf]

Supplementary information

Applied Microbiology and biotechnology

Trans-nuclei CRISPR/Cas9: Safe approach for genome editing in the edible mushroom excluding foreign DNA sequences

Daishiro Koshi[a], Junko Sugano[a], Fuga Yamasaki[a], Moriyuki Kawauchi[a], Takehito Nakazawa [a], Minji Oh[b], Yoichi Honda[a, *]

[a] Graduate School of Agriculture, Kyoto University, Kitashirakawaoiwakecho, Sakyo-ku, Kyoto 606-8502, Japan

[b] Mushroom Division, National Institute of Horticultural and Herbal Science, Rural Development Administration, Bisanro 92, Eumseong, Chungcheongbuk 27709, Republic of Korea

*** Corresponding author:** Yoichi Honda

Graduate School of Agriculture, Kyoto University, Kitashirakawaoiwakecho, Sakyo-ku, Kyoto 606–8502, Japan

Email address: honda.yoichi.5n@kyoto-u.ac.jp

Phone number: +81-75-753-6463 Fax number: +81-75-753-6471

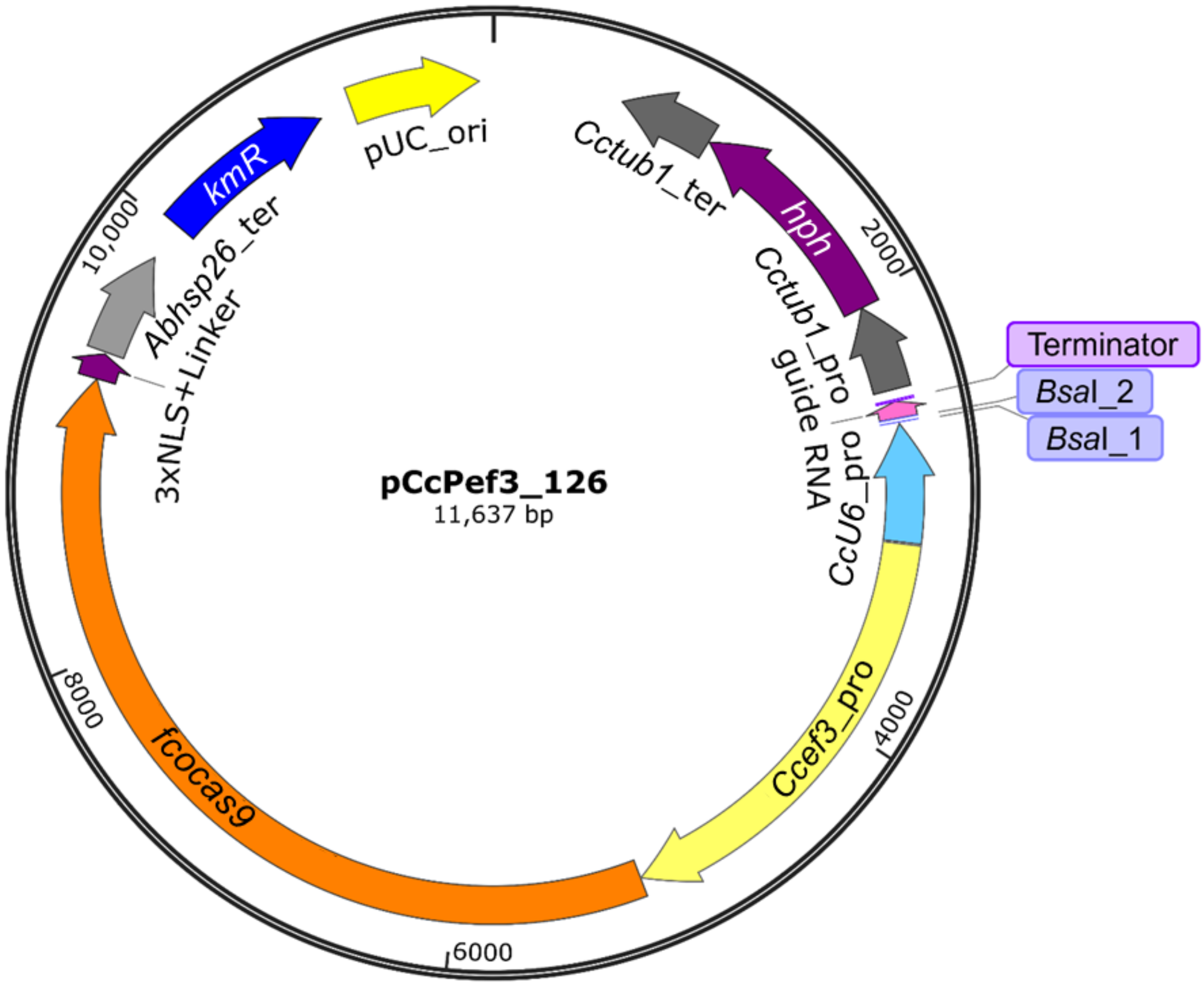

Figure S1 A plasmid map of pCcPef3-126. pCcPef3-pyrGsg1 and pCcPef3-pyrGsg2 were constructed by inserting gRNA-coding sequences, *pyrG*sg1 and *pyrG*sg2, respectively. pUC_ori: Origin for *Escherichia coli*, *kmR*: Kanamycin resistance gene, *Abhsp26*_ter: Terminator from *hsp26* gene in *Agaricus bisporus*. NLS: Nuclear localization signal, *fcocas9*: Fungal and plant codon optimized Cas9 (Sugano et al. 2017), *Ccef3*_pro: Promoter from *ef3* gene in *Coprinopsis cinerea*, *CcU6*_pro: *U6* promoter from *C. cinerea*, *Cctub1*_pro or ter: Promoter or terminator from *tub1* gene in *C. cinerea*, *hph*: Hygromycin phosphotransferase, *Bsa*I_1 or 2: *Bsa*I cutting site.

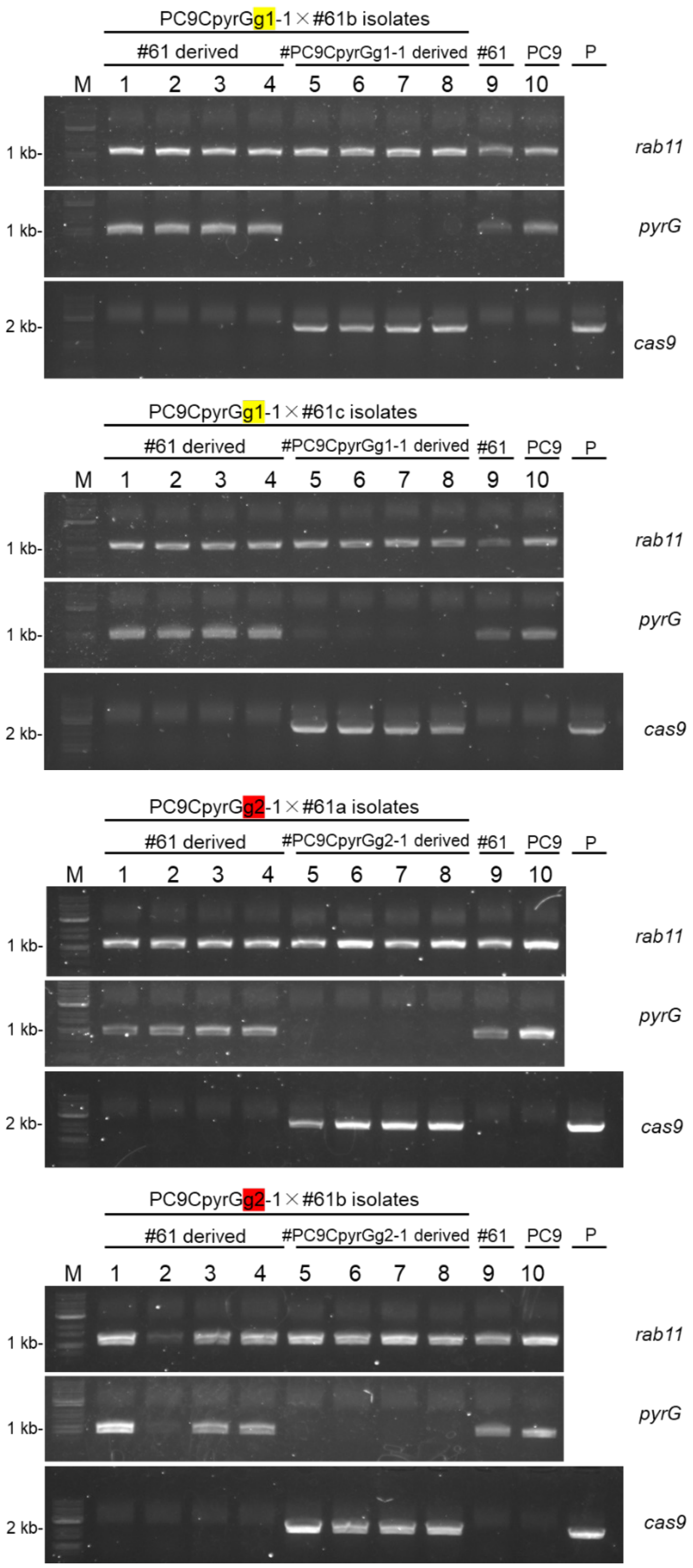

Figure S2 Whole gel electrophoresis data of genomic PCR confirming *cas9* fragment amplification in the isolated monokaryotic strains. Lane M: 1 kb ladder marker; Lanes 1-4: Monokaryotic strains with #61 (recipient) morphologies; Lanes 5-8: Monokaryotic strains with PC9 (donor PC9CpyrGg1/2-1) morphologies; Lane 9: Wild-type #61 strain; Lane 10: PC9 strain; Lane P: Plasmid control.

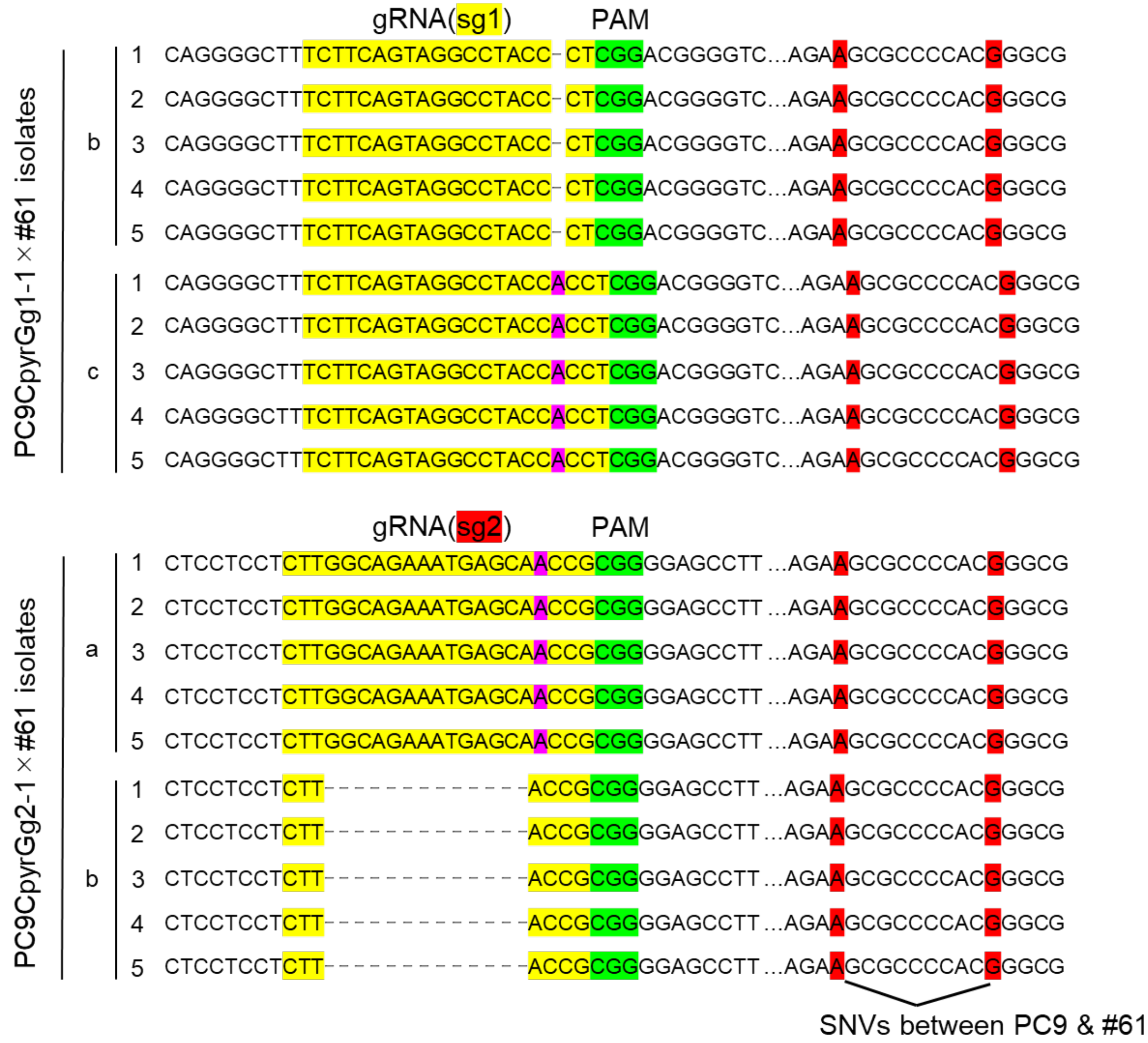

Figure S3 Sequence analysis results of the isolated monokaryotic strains with recipient-derived nuclei. The shaded regions in the nucleotide sequence indicate yellow for the target sites of gRNA, green for protospacer adjacent motif (PAM) sequences, and red for single nucleotide variants (SNVs) between PC9 and #61 strains.

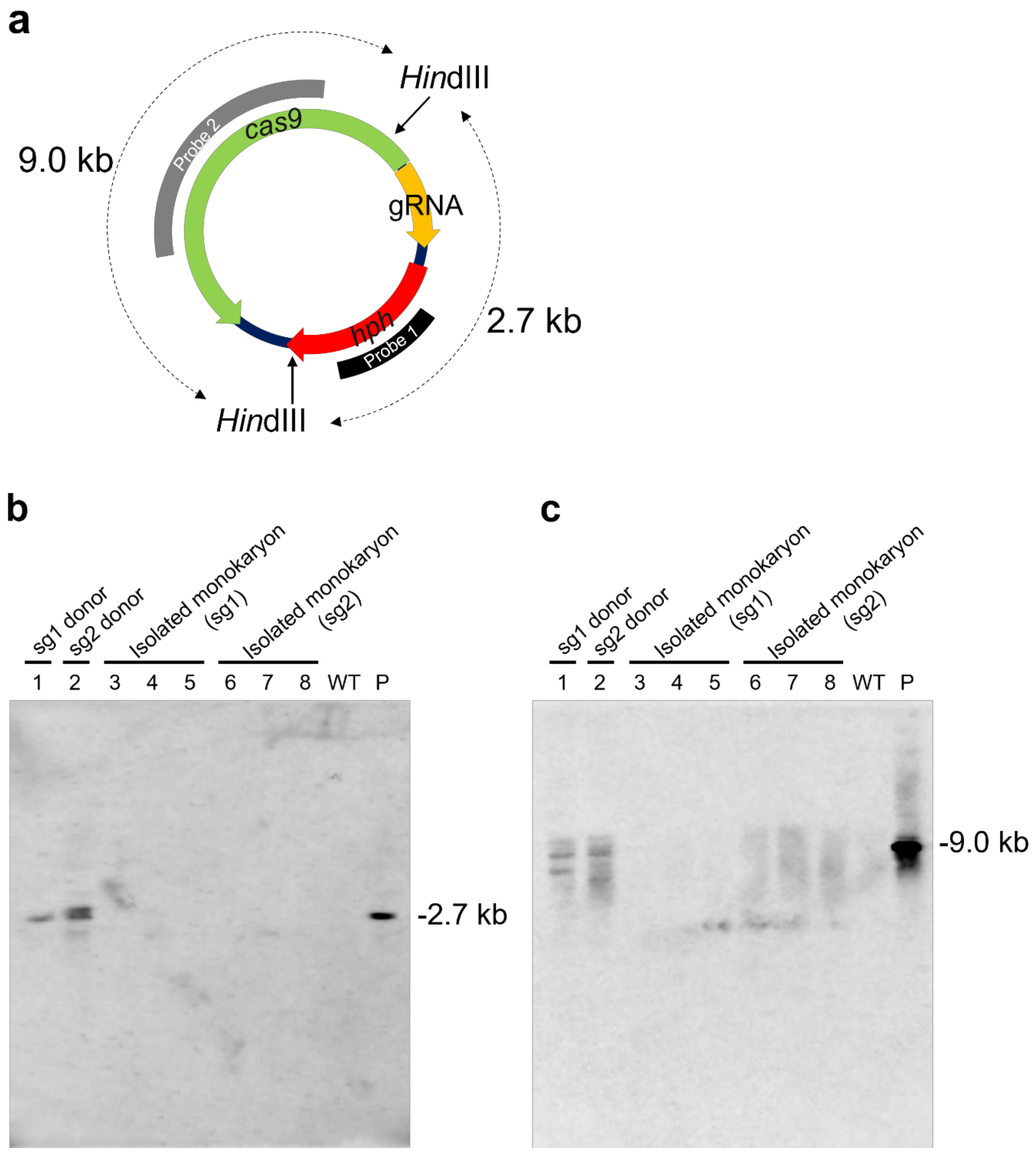

Figure S4 Southern blotting analysis of monokaryotic strains with nuclei derived from #61. (a) Image of pCcPef3-pyrGsg1/sg2 and restriction sites for *Hin*dIII. Two thick black/gray lines above the plasmid indicate the genomic fragments used as probes. (b) Results of Southern blotting analyses using the black probe. (c) Results of Southern blotting analyses using the gray probe. Lane 1, PC9CpyrGg1-1; lane 2, PC9CpyrGg2-1; lanes 3-5, Recipient monokaryotic strains derived from PC9CpyrGg1-1x#61-2; lanes 6-8, Monokaryotic strains with nuclei derived from PC9CpyrGg2-1; lane WT, #61; Lane P, *Hin*dIII-digested pCcPef3-pyrGsg2.

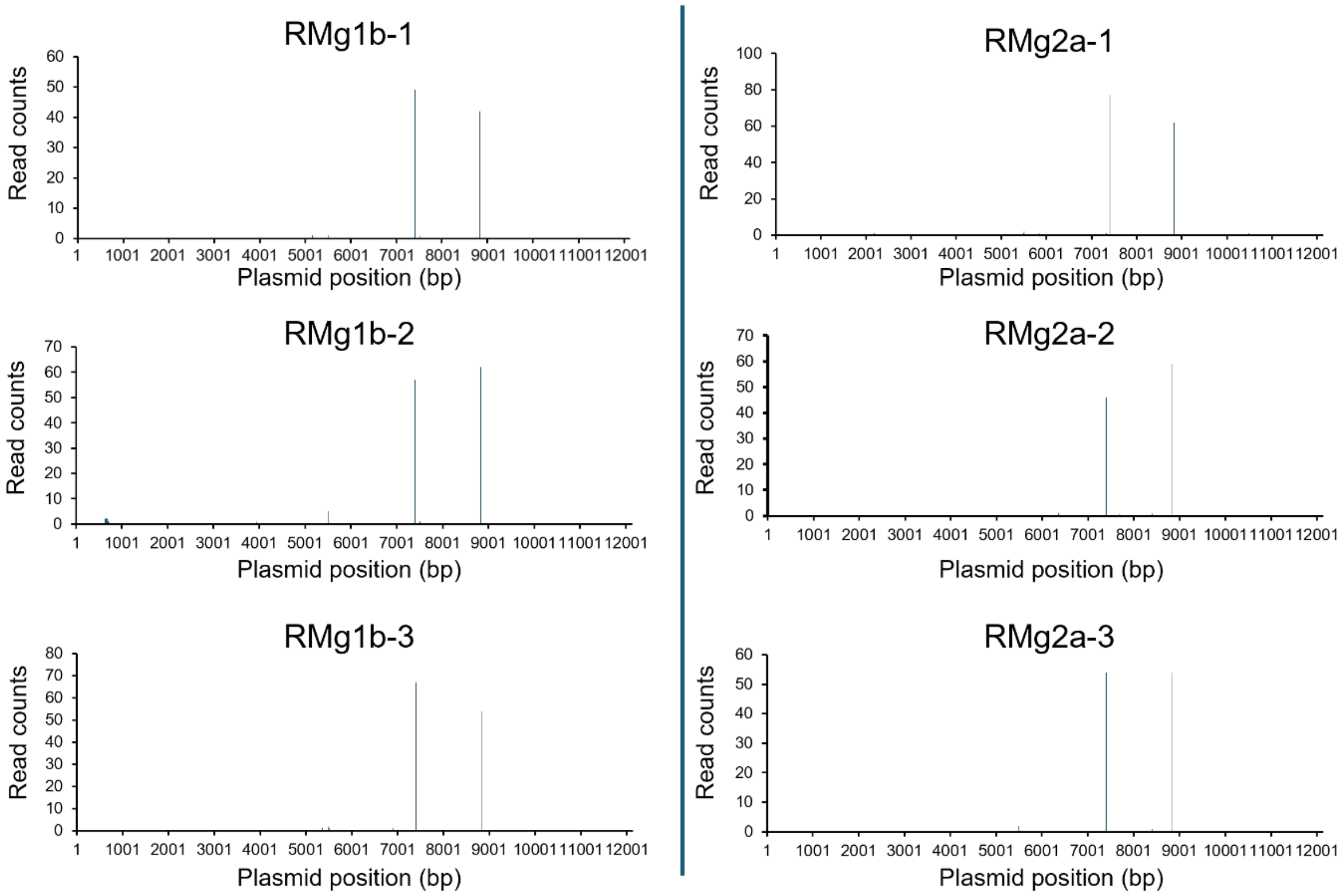

Figure S5 All read count data of the identical 20-mer detection analysis of monokaryotic strains with nuclei derived from #61 (RMg1b-1, 2, 3, RMg2a-1, 2, 3). The y-axis show the read counts of a 20-mer at each nucleotide position. The x-axis indicates the nucleotide positions on a vector sequence.

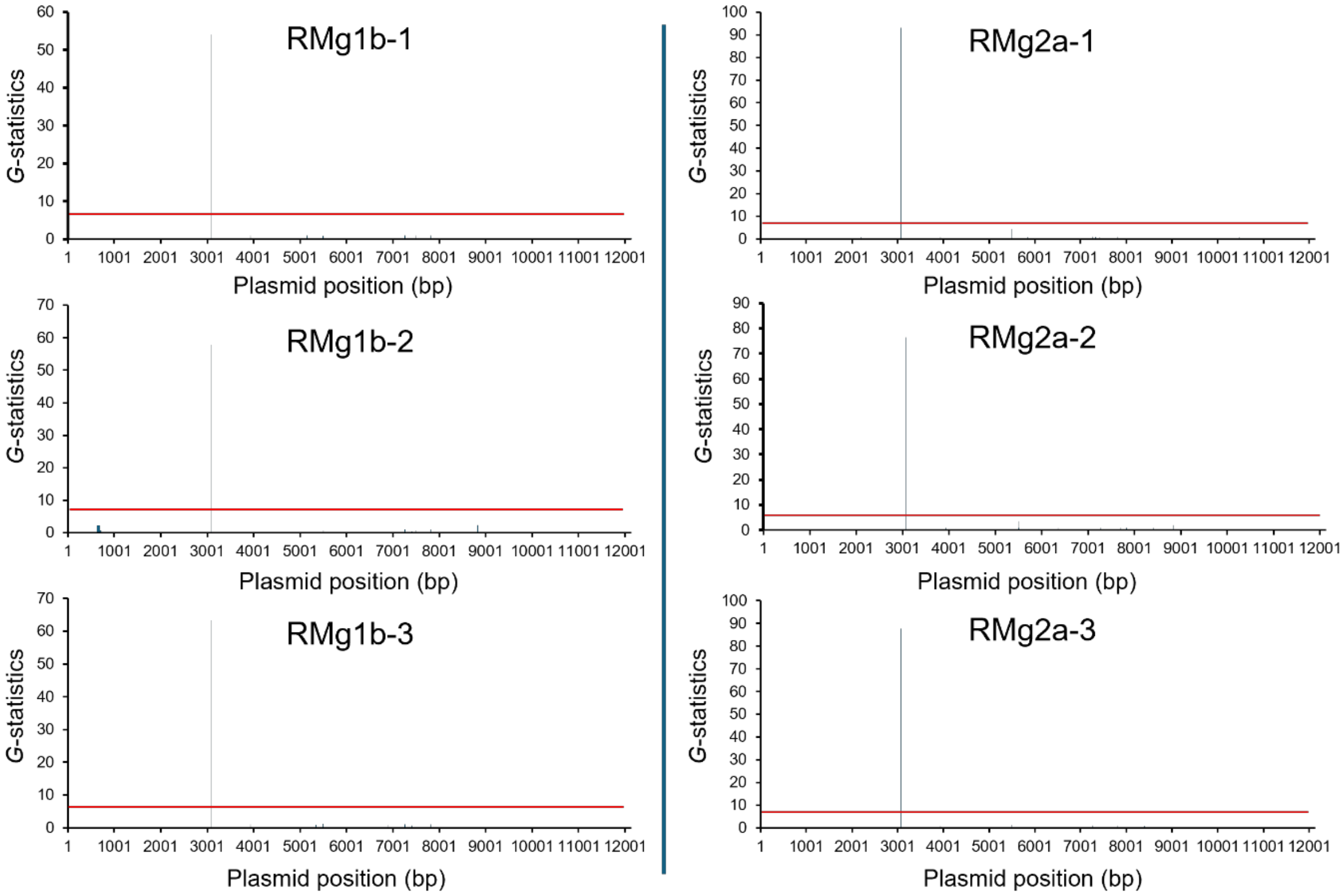

Figure S6 All *G*-test data of the identical 20-mer detection analysis of monokaryotic strains with nuclei derived from #61 (RMg1b-1, 2, 3, RMg2a-1, 2, 3). The y-axis show the *G*-statistic of a 20-mer at each nucleotide position. The x-axis indicates the nucleotide positions on a vector sequence. The red horizontal line corresponds to the 1% significance level (*G*-values > 6.634).

Table S1 *P. ostreatus* strains used in this study

Strain	Description	Source
PC9	A2B1/5-FOA sensitive (Nakazawa et al. 2016)	CECT20311; Larraya et al. (1999)
#61	A61B61/5-FOA sensitive	NBRC116971; this study
PC9CpyrGg1-1	A2B1/a 5-FOA-resistant strain obtained after introducing pCcPef3-126-pyrGsg1	This study
PC9CpyrGg1-2	A2B1/a 5-FOA-resistant strain obtained after introducing pCcPef3-126-pyrGsg1	This study
PC9CpyrGg2-1	A2B1/a 5-FOA-resistant strain obtained after introducing pCcPef3-126-pyrGsg2	This study
PC9CpyrGg2-2	A2B1/a 5-FOA-resistant strain obtained after introducing pCcPef3-126-pyrGsg2	This study

Table S2 Primers used in this study

Name	Sequence (5'-3')
KK33	GACGAGCTGTACAAGATGGCTGAAGGGAGCAACTACG
KK34	GCTGTTAACTTGTGACTAGCAACATTTACCACCCTGGTTC
TB41	GATTGTCTTCAGTAGGCCTACCCCT
TB42	AAACAGGGGTAGGCCTACTGAAGAC
TB43	GATTGCTTGGCAGAAATGAGCACCG
TB44	AAACCGGTGCTCATTTCTGCCAAGC
TB53	CGCCGCTTGTAGGAAACACAG
TK94	CAAAGATAGGTTCGTCGTCCGTTAG
TK161	GACGATGTTGACCTGAGGCA
TK162	TTCGACCAGTCCAAGAACGG
TK203	ACTCACCGCGACGTCTGTCGAGAAG
TK204	CTATTCCTTTGCCCTCGGACGAGTGC

Table S3 Summary of NGS data acquisition

Sample	Accession No. [a]	Number of reads	Mapped read bases (Mb)	Coverage [b] (x)
#61 (Wild type)	DRR584960	15,957,966	2,268	64.73
RMg1b-1	DRR584954	15,146,224	2,149	61.35
RMg1b-2	DRR584955	16,612,378	2,360	67.37
RMg1b-3	DRR584956	18,951,286	2,690	76.80
RMg2a-1	DRR584957	21,500,218	3,049	87.04
RMg2a-2	DRR584958	16,245,972	2,304	65.76
RMg2a-3	DRR584959	19,690,250	2,792	79.70

[a] Sequence data are available in the DDBJ BioSample database under accession number listed.

[b] Number of bases of reference: 350 Mb

References

Larraya LM, Pérez G, Peñas MM, Baars JJP, Mikosch TSP, Pisabarro AG, Ramírez L (1999) Molecular karyotype of the white rot fungus *Pleurotus ostreatus*. Appl Environ Microbiol 65:3413–3417. https://doi.org/10.1128/AEM.65.8.3413-3417.1999

Nakazawa T, Tsuzuki M, Irie T, Sakamoto M, Honda Y (2016) Marker recycling via 5-fluoroorotic acid and 5-fluorocytosine counter-selection in the white-rot agaricomycete *Pleurotus ostreatus*. Fungal Biol 120:1146–1155. https://doi.org/10.1016/j.funbio.2016.06.011

Sugano SS, Suzuki H, Shimokita E, Chiba H, Noji S, Osakabe Y, Osakabe K (2017) Genome editing in the mushroom-forming basidiomycete *Coprinopsis cinerea*, optimized by a high-throughput transformation system. Sci Rep 7:1260. https://doi.org/10.1038/s41598-017-00883-5
